# 

**Published:** 1830-05

**Authors:** 


### MONTHLY LIST OF MEDICAL BOOKS.

[*Medical Works cannot be entered on this List except a copy be sent for the purpose; the titles of Books having frequently been transmitted to us, as published, which have not appeared for weeks, or even months, after.*]

The Influence of Climate in the Prevention and Cure of Chronic Diseases, more particularly of the Chest and Digestive Organs: comprising an Account of the principal Places resorted to by Invalids in England, the South of Europe, &c.; a comparative Estimate of their respective Merits in particular Diseases; and General Directions for Invalids while travelling and residing abroad. With an Appendix, containing a Series of Tables on Climate. By JAMES CLARK, M.D. &c. SECOND EDITION, enlarged.—8vo. pp. 400. Underwood, London, 1830.

Addresses delivered on various Public Occasions, by JOHN GODMAN, M.D. &c. Containing a brief Explanation of the Injurious Effects of tight Lacing upon the Organs and Functions of Respiration, Circulation, Digestion, &c. —8vo. pp. 194. Carey, Philadelphia, 1829.

Medical Botany, No. XL. April 1830. By JOHN STEPHENSON, M.D. F.L.S. &c. and J. M. CHURCHILL, F.L.S. &c.

This Number contains excellent plates and descriptions of the Scilla Maritima, Pimpinella Anisum, Amyris Gileadensis, Copaifera Officinalis, and Papaver Somniferum.

A Practical Essay on the Disease generally known under the Denomination of Delirium Tremens; written principally with a View to elucidate its Division into distinct Stages, and hence to simplify its Method of Cure. By ANDREW BLAKE, M.D. &c.—Pp. 68. Burgess and Hill, London, 1830.

A Treatise on Deformities; exhibiting a concise View of the Nature and Treatment of the principal Distortions and Contractions of the Limbs, Joints, and Spine. Illustrated with Plates and Woodcuts. By LIONEL J. BEALE, Surgeon.—8vo. pp. 248. Wilson, London, 1830.

A Letter to WILLIAM LAWRENCE, Esq. F.R.S. on the Nature and Causes of Intellectual Life and the Mind. By WILLIAM ADDISON, Member of the Royal College of Surgeons in London.—8vo. Underwoods, London; and J. Knib, Worcester, 1830.

A Manual of Descriptive Anatomy; illustrated by Lithographic Plates. By M. CLOQUET. Translated from the French, by THOMAS KING, late House Surgeon to the Hôtel Dieu, and Lecturer on Anatomy at the Aldersgate-street Medical School. Part I.—Dumus and Co. Leicester square, London, 1830.

Surgical Observations on the more important Diseases of the Mucous Canals of the Body; being a second Edition of the Author's Treatise on Stricture of the Urethra. To which are added, Practical Observations on Contraction of the Œsophagus and Rectum; an Essay on the Diagnosis of Hernial and other Tumors in the Groin; with Remarks on Tracheotomy, as connected with the Treatment of Chronic Laryngitis. By G. MACILWAIN, Surgeon to the Finsbury Dispensary, &c.—8vo. pp. 337. Longman, London, 1830.

Analytical Anatomy. Great Sympathetic Nerve. By P. J. MANEC, D.M.P. &c. Folio Plate.—Dumus and Co. Leicester square.

Immense labour must have been bestowed upon this plate, but we very unwillingly state that we cannot conscientiously recommend it as a study. From the number of minute parts that are contained in it, numerous letters and figures of reference are required, which are necessarily so close together as to render the whole complicated and perplexing. The task of making out the different parts would be very laborious, and we fear almost impracticable.

An Introductory Lecture to the Theory and Practice of Midwifery; being an Historical Account of that subject, publicly delivered in London, October 4th, 1829. By THOMAS GREENING, M.D. &c.—London, 1830.

A Clinical Report Of the Royal Dispensary for Diseases of the Ear; with Remarks on the Objects and Utility of the Institution. By J. H. CURTIS, Esq. &c.—8vo. pp. 53. Longman, London, 1830.

### NOTICE TO CORRESPONDENTS.

*The Editor informs* Dr. A. *that there is no work which treats exclusively of the subject.* COLLINSON *on the Law of Lunacy, and* WRIGHT'S *Reports of Lord Eldon's Judgments and Decisions, contain some interesting information upon it.* FONBLANQUE *and* PARIS, *and most of the works on Medical Jurisprudence, refer to it. The information scattered through the general Chancery Reports upon the point referred to would be valuable, but is scarcely accessible to the medical inquirer.*

*The Editor regrets that he cannot comply with the request of* Mr. F. N.

*The case received from Gloucester is too extraordinary to be published, without being authenticated by the name of the author of the communication.*

*Communications have been received from* Dr. C. J. ROBERTS, Mr. J. FOOTE, jun., *and* Mr. J. RAINES.

